# Verbal and Visuospatial Working Memory Performance During the CO_2_ Challenge Model of Anxiety

**DOI:** 10.1002/hup.70051

**Published:** 2026-06-21

**Authors:** Warren Dunger, Marc Edwards, Alex Board, Matthew Garner

**Affiliations:** ^1^ School of Psychology University of Southampton Southampton UK

**Keywords:** anxiety, CO_2_ Challenge, neuropsychological assessment, working memory

## Abstract

**Objective:**

Anxiety influences working memory performance, but the effects on clinical neuropsychological assessments of working memory are not well known.

**Methods:**

We examined the effect of anxiety, induced using the 7.5% carbon dioxide model of anxiety on standardized clinical neuropsychological tests of working memory and executive function in a single‐blind, placebo‐controlled, randomized, crossover within‐subjects design.

**Results:**

The CO_2_‐challenge reduced spatial working memory performance and verbal working memory performance accuracy when task demands were high. The CO_2_‐challenge increased effort and reduced processing efficiency across all verbal and spatial working memory tasks.

**Conclusions:**

The CO_2_‐challenge resulted in significant reductions in working memory performance and processing efficiency. These results encourage the routine assessment of anxiety when administering and interpreting neuropsychological measures of working memory function in clinical practice.

## Graphical abstract

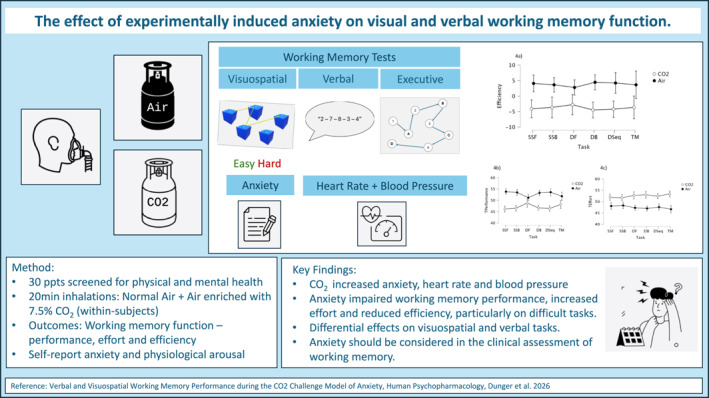

## 1 Introduction

Neuropsychological factors that influence cognitive functioning can be broadly classed into ‘primary’ and ‘secondary’ influences (Arnett 2013). Primary (i.e., organic) influences include direct insult/injury to the brain (e.g., traumatic head injury, stroke), whereas secondary (i.e., functional) influences include fatigue, pain, mood and anxiety. Anxiety is widely reported by those attending clinical appointments in primary care (Wiegner et al. 2015) and secondary/tertiary clinical services (Walker et al. 2021). It can affect patients' description of their symptoms (Granot and Ferber 2005) and adherence/clinical response to treatment (Santana and Fontenelle 2011; Suls and Howren 2012). During a neuropsychological assessment in clinical practice, anxiety is often reported by clients who are concerned about their diagnosis and prognosis (Dorenkamp and Vik 2018). However, the effects of anxiety on cognitive performance measures that are used in routine neuropsychological assessment are not well understood.

Cognitive models predict that anxiety reduces attentional control in working memory and increases distractibility (Derakshan and Eysenck 2009; M. Eysenck and Derakshan 2011) with deficits in processing efficiency (time/effort) observed before effects on performance effectiveness (e.g., accuracy). Specifically, anxiety is considered to impact the efficiency of executive functioning (inhibition, shifting, and updating) which is mediated by both ‘top‐down’ (goal‐directed attention) and ‘bottom‐up’ (stimulus‐driven attention). When cognitive tasks require attentional control, anxiety can reduce processing efficiency due to increased top‐down and reactive attention networks (M. W. Eysenck et al. 2023), which result in measurable performance deficits, despite efforts to compensate. Cognitive and physiological symptoms of anxiety may also have different effects on cognition; anxious‐apprehension (i.e., verbal worry about future negative outcomes) is likely to interfere with verbal working memory due to competition in the dorsal, medial and left ventral prefrontal regions (D'Esposito et al. 1998; Engels et al. 2007; Kalisch et al. 2006; Paulesu et al. 2010) and anxious‐arousal (i.e., the physiological/somatic experience of anxiety) affects spatial working memory due to competition for resources in the right prefrontal and posterior‐parietal cortex (Balderston et al. 2017; Lavric et al. 2003; Murphy et al. 2003; Nee et al. 2013).

The cognitive effects of anxious apprehension and arousal may differ with task demands; while high levels of autonomic arousal may affect performance across high and low load tasks, the effects of anxious apprehension may be over‐ridden when task demands and compensatory effort increase (Balderston et al. 2016; K. Vytal et al. 2013). Few studies have directly investigated the effects of anxiety on mental effort in clinical assessments, despite evidence that effort increases with anxiety on non‐clinical cognitive tasks (Edwards et al. 2015, 2016; Hadwin et al. 2005; Kalisch et al. 2006). Early studies of digit span performance observed detrimental effects of anxiety in healthy young adults (Hodges and Spielberger 1969; Hodges and Durham 1972). Furthermore, the use of clinical neuropsychological assessments may be beneficial due to their widespread availability, standardisation and known psychometric properties (Coy et al. 2011), and to determine the ‘clinical’ significance of anxiety in clinical assessment and practice. Correlational and between‐subjects designs often do not adequately consider individual differences in working memory ability (Daily et al. 2001; Mella et al. 2016), nor susceptibility to mood‐induction procedures (Gomez et al. 2000; Scherrer and Dobson 2009), and mood induction procedures may elicit only transient effects that do not sustain throughout the experimental/clinical assessment (see Moran 2016 for a review).

The inhalation of air enriched with 7.5% carbon dioxide (CO_2_) is an established anxiety‐induction procedure which produces a temporary generalized anxiety state that demonstrates some chronic symptoms that are resolved after cessation of the 20‐min inhalation. The procedure allows performance measures (e.g., cognitive tasks) to be completed during sustained periods of elevated anxiety (Bailey et al. 2005). Studies using this approach have found increased reports of anxiety as well as increases in physiological arousal such as heart rate, blood pressure and respiratory rate (Bailey et al. 2005, 2011; Gillan et al. 2021; Gnacek et al. 2025; Pinkney et al. 2014; Poma et al. 2005).

Between‐group studies comparing the effects of 7.5% CO_2_ versus air inhalation on cognition, provide evidence that the CO_2_ challenge increases attention to threat distractors (Garner et al. 2011) and increases hypervigilance (alerting and orienting attention network function; Garner et al. 2012). To date, only a single study has used this induction procedure to investigate anxiety and working memory measures from the CANTAB (Savulich et al. 2019). Results provided evidence that CO_2_ inhalation impaired executive measures of cognitive flexibility and working memory. However, a measure of verbal working memory was not included to test domain‐specific predictions and only a smaller subset of participants completed the spatial working memory task.

Here we used a single‐blind, placebo‐controlled, randomized, crossover within‐subjects design to examine the effects of 7.5% CO_2_ induced‐anxiety on working memory performance using standardised neuropsychological assessments of auditory‐verbal (Digit Span) and visuospatial (Spatial Span) working memory, which are commonly used in clinical practice (Camera et al. 2000; Rabin et al. 2005). We examined the effects of anxiety on both working memory performance (i.e., total score for each measure), effort, and efficiency (i.e., the relationship between performance and invested mental effort) and under conditions of low cognitive load (forwards span) and high load (backwards span). The effect of anxiety on executive processes (set‐shifting) was also measured using the Trail‐Making test, which is routinely used in clinical neuropsychology practice. We predicted that CO_2_ inhalation will replicate previous findings and increase subjective anxiety, heart rate and blood pressure. Furthermore, we predicted that CO_2_ inhalation will reduce performance (effectiveness) and increase effort on spatial and verbal working memory tasks and corresponding measures of performance efficiency. We predicted that the effects of anxiety on processing efficiency would be most pronounced on high load WM tasks where anxiety prevented compensatory effort required to perform tasks effectively.

## 2 Method

### 2.1 Participants

Thirty‐six healthy participants were recruited from the community. Five participants withdrew after the first test session (5 CO_2_, 0 air) and one participant was removed due to English‐language difficulties. The final sample comprised 30 healthy young adults (25 female sex) between the ages of 18–26 years (*M* = 19.8 years, SD = 2.3). A‐priori power size calculations were based on large effect sizes (within‐subjects) of induced anxiety on working memory/cognitive control in CO_2_‐induced anxiety manipulations (Garner et al. 2011, *np* ^2^ = 0.152) and expectancy manipulations of anxiety (K. Vytal et al. 2012, *n* ^2^ 0.13). Consequently, we calculated a conservative sample size = 20+ participants was required, assuming power at 80% and alpha = 0.05 to reveal effects across multiple task conditions. Participants received course credit or payment for participation. The study received ethical approval from the University of Southampton Research Ethics and Governance Committee (ERGO 18175).

Consistent with previous 7.5% CO_2_ challenge studies, participants completed a telephone pre‐screening interview based on DSM‐5 criteria (Mini International Neuropsychiatric Interview; Sheehan et al. 1998). Exclusion criteria included recent use of medication (past 8 weeks except aspirin, paracetamol, contraceptive pill), pregnancy, history of asthma/respiratory illness, high blood pressure (> 140/90 mmHg), cardiovascular disease, migraines, current or lifetime history of psychiatric illness (including family history of panic attacks), smoker, under‐or over‐weight (body‐mass index < 18 or 28 >), current or past drug or alcohol dependence and recent use of illicit drugs, or alcohol (breath test). Participants completed standardized measures of trait anxiety (STAI‐Trait; Spielberger et al. 1983) and trait worry (Penn State Worry Questionnaire; Meyer et al. 1990). Levels of trait anxiety (*M* = 35.17, SD = 8.00) and worry (*M* = 43.47, SD = 11.25; PSWQ) were comparable to those reported in healthy samples following similar screening protocols (e.g., Garner et al. 2011; Savulich et al. 2019).

### 2.2 Materials and Procedure

The study used a single‐blind mixed multi‐factorial design with each participant attending two testing sessions in person following the telephone screen. All participants provided informed written consent prior to taking part. During the first session participants were randomly assigned to either the experimental (CO_2_) or control inhalation condition (normal air), completing the alternate condition between 7 and 14 days after session 1 (*M* = 10.7 days, SD = 5.9). This variable interval was due to participant availability, but ensured at least a 7 day interval to reduce carry‐over effects from session 1. Inhalation condition order was counterbalanced. Participants were blind to the condition, although 93.3% (*n* = 28) correctly guessed the order at debrief.

Physiological measures of anxiety (heart rate and blood pressure using Omron‐M6, arm‐collar; Medisave‐UK) and questionnaire measures of state anxiety (modified GAD‐7; Spitzer et al. 2006 & STAI‐State; Spielberger et al. 1983) were taken at baseline and immediately after the 20 min inhalation of either air enriched with 7.5% CO_2_ (21% O_2_, balance N_2_) or normal air via oro‐nasal face mask. Anxiety symptoms were measured using a modified version of the GAD‐7 questionnaire (Spitzer et al. 2006). The GAD‐7 is a 7‐item questionnaire used to screen for generalized anxiety disorder (GAD) and assess its severity. In this modified version, participants respond indicating how often they have been bothered by common generalized anxiety symptoms ‘During the last 5 min’. Each item is scored on a visual analogue scale (VAS) ranging from 0 ‘not at all’ to 100 ‘all of the time’. This version of the GAD‐7 VAS has previously shown good sensitivity to changes in anxiety during acute anxiety challenges (Huneke et al. 2020, 2022, 2024; Özhan et al. 2026).

Two minutes after the start of the inhalation, participants completed the standard paper‐based neuropsychological assessments in fixed order: (i) executive set‐shifting task (Trail‐Making from the Delis‐Kaplin Executive Function System; Delis et al. 2001), (ii) verbal working memory (Forwards, Backwards and Sequencing Digit Span subtests from the Wechsler Adult Intelligence Scale–Fourth Edition; Wechsler 2008), and (iii) spatial working memory (Forwards and Backwards Spatial Span subtest from the Wechsler Memory Scale–Third Edition; Wechsler 1997).

Participants were asked to rate ‘mental effort’ after completing each subtest (Rating Scale for Mental Effort; Zijlstra 1993; Range 0–100). Tests were administered by either a trainee clinical psychologist or research assistant with appropriate training. The approximate start times for each cognitive test/subtest during the 20 min (air inhalation) were as follows: Trail Making (2 min—visual search, 3 min—number sequencing, 4 min—letter sequencing, 5 min—number‐letter switching, 8 min—motor speed), Digit Span (9 min—forwards, 11 min—backwards, 13 min—sequencing), and Spatial Span (15 min—forwards, 18 min—backwards). Test order intentionally began with the non‐verbal executive switching task and control conditions to allow time for the cumulative effects of CO_2_ inhalation to become apparent within 3 min. Previous research has found physiological changes (e.g., galvanic skin response and respiration rate) peaking within approximately 3 min in response to the CO_2_ challenge (Bailey et al. 2005; Gnacek et al. 2025).

### 2.3 Data Preparation

The total number of successfully repeated number strings for each condition (e.g., Digit Span Forwards) before meeting the discontinue criteria was used to measure working memory performance (as per test manual). In contrast, the Trail Making Performance scores reflect task completion speeds (seconds) and are analysed separately. To measure the processing efficiency of working memory, mental effort ratings and working memory performance scores were converted to a common standardised metric (T‐Scores; *M* = 50, SD = 10; de Beurs et al. 2022), using participants performance across the two inhalation conditions. These were weighted against each other using the mental efficiency formula from Paas and Merriënboer (1993):

$$Processing\;Efficiency=\frac{\left(WM\;Performace-Mental\;Effort\right)}{\sqrt{2}}$$

Large positive scores reflect improved processing efficiency (i.e., high levels of performance achieved with low levels of effort), whereas negative scores reflect poor efficiency (i.e., low levels of performance despite high levels of effort).

## 3 Results

### 3.1 Manipulation Check: Effect of CO_2_ on Anxiety and Cardiovascular Function

**FIGURE 1:**
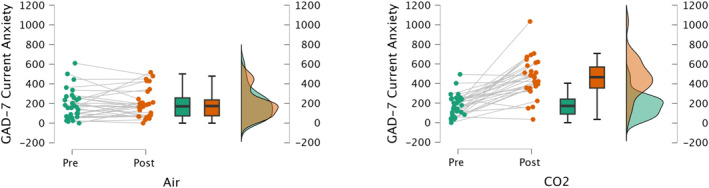
GAD‐7 Anxiety before and after the inhalation of air (left panel) and CO_2_ (right panel). Box plots display median, lower quartile, upper quartile and range of non‐outlier values (< 1.5 × IQR) for each time × inhalation condition.

Effects on psychological and physiological measures of state anxiety were examined in 2 (Inhalation: Air vs. CO_2_) × 2 (Time: Pre vs. Post) ANOVA. Consistent with previous studies the inhalation of 7.5% CO_2_ increased state anxiety, heart rate and systolic blood pressure (see Table 1 and Figure 1).

**TABLE 1:** Descriptive statistics for psychological and physiological measures of state anxiety.

	Normal air						CO_2_						Inhalation * time F(1,29)
	Pre			Post			Pre			Post			
	*M*	SD	*α*	*M*	SD	*α*	*M*	SD	*α*	*M*	SD	*α*	
GAD‐7	192.0^a^	148.6	0.89	199.2^a^	143.7	0.93	174.9^a^	112.3	0.81	458.7^b^	196.0	0.87	40.425, *p* < 0.001, *n* ^*2*^ = 0.187
STAI‐S	32.4^a^	9.7	0.92	37.6^a^	9.4	0.89	33.6^a^	8.5	0.87	50.4^b^	10.7	0.87	17.243, *p* < 0.001, *n* ^*2*^ = 0.079
HR	77.8^a^	15.3	—	72.1^a^	9.5	—	76.7^a^	15.6	—	92.9^b^	17.0	—	25.919, *p* < 0.001, *n* ^*2*^ = 0.0189
sBP	130.4	15.0	—	121.8^a^	12.3	—	130.6	14.2	—	136.1^b^	14.8	—	21.341, *p* < 0.001, *n* ^*2*^ = 0.110
dBP	73.4	9.3	—	70.6	8.5	—	76.1	10.7	—	76.3	7.4	—	2.114, *p* = 0.157, *n* ^*2*^ = 0.018

*Note:* Within each variable (row), the values with different superscripts are significantly different from each other: *p* < 0.017 (Bonferroni correction applied). *α = Chronbach's alpha.*

Abbreviations: dBP = Diastolic blood pressure; GAD‐7 = Generalised Anxiety Disorder Assessment‐7; HR = Heart Rate; sBP = Systolic blood pressure; STAI‐S = State‐Trait Anxiety Inventory‐State.

### 3.2 Working Memory Performance

**FIGURE 2:**
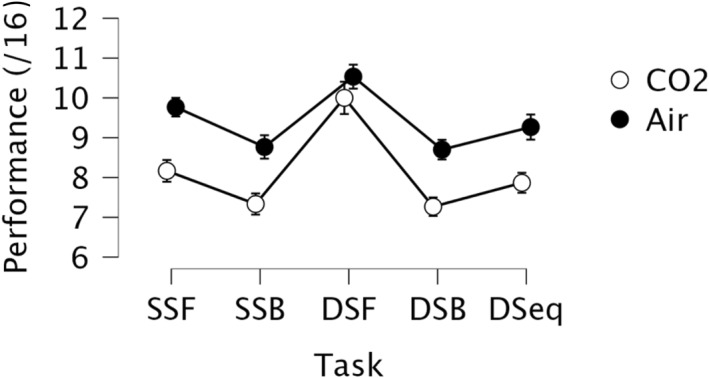
Spatial and verbal performance during CO_2_ inhalation and air inhalation (scores/16).

Repeated‐measures ANOVA explored the effect of inhalation on measures of spatial, verbal and executive working memory performance. Performance scores are shown in Table 2 and Figure 2.

**TABLE 2:** Descriptive statistics for performance (P) and mental effort (ME) ratings for working memory measures.

Variable^a^	Normal air				CO_2_			
	*M*(P)	SD	*M*(ME)	RSME SD	*M*(P)	SD	*M*(ME)	RSME SD
Spatial span forwards (SSF)	9.77	(1.94)	64.30	(24.25)	8.17	(1.93)	73.40	(21.94)
Spatial span backwards (SSB)	8.77	(2.08)	69.60	(28.27)	7.33	(1.81)	78.43	(23.87)
Spatial span total	18.43	(3.70)	—	—	15.60	(3.19)	—	—
Digit span forwards (DF)	10.53	(2.00)	56.17	(19.81)	10.00	(2.46)	68.87	(24.90)
Digit span backwards (DB)	8.70	(2.18)	66.47	(20.15)	7.27	(1.93)	79.77	(22.30)
Digit span sequencing (DSeq)	9.27	(1.95)	71.30	(26.79)	7.87	(1.72)	83.17	(21.27)
Digit span total	28.50	(4.65)	—	—	25.13	(4.86)	—	—
Trail‐making (condition 4)	57.90	(17.05)	52.13	(23.51)	64.23	(17.86)	68.50	(22.80)

*Note:* Number‐Letter Switching (Condition 4) of Trail‐Making with lower scores representing faster completion times.

Abbreviation: RSME = Rating Scale for Mental Effort (0–100).

^a^ Test score range 0–16 for SSF, SSB, DF, DB, DSeq.

To compare performance across spatial and verbal tasks we entered performance scores (/16 max) in a 2 (Inhalation) × 5 (Task) ANOVA. The effect of CO_2_ (vs. air) differed across tasks [Task × Inhalation, F(4,116) = 2.543, *p* = 0.043, *np* ^2^ = 0.081]. Regarding spatial working memory, CO_2_ inhalation impaired performance on Spatial Span Forwards (SSF), *F*(1,29) = 37.622, *p* < 0.001, *n* ^2^ = 0.565, and Spatial Span Backwards (SSB), *F*(1,29) = 24.362, *p* < 0.001, *n* ^2^ = 0.457 tasks (Figure 2). Regarding verbal working memory tasks, CO_2_ inhalation impaired performance on the Digit Span Backwards (DB), *F*(1,29) = 36.206, *p* < 0.001, *np* ^2^ = 0.555 and Digit Span Sequencing (DSeq), *F*(1,29) = 199.234, *p* < 0.001, *d* = 0.873 tasks, but did not impact Digit Span Forwards (DF) performance *F*(1,29) = 2.303, *p* = 0.140, *np* ^2^ = 0.074 (Figure 2).

Repeated‐measures MANOVA did not provide evidence that CO_2_ inhalation impacted performance across Trail‐Making (TM) conditions, *F*(5,25) = 1.031, *p* = 0.421, *np* ^2^ = 0.171 (including the primary measure condition 4: number‐letter switching; Table 2).

### 3.3 Mental Effort

**FIGURE 3:**
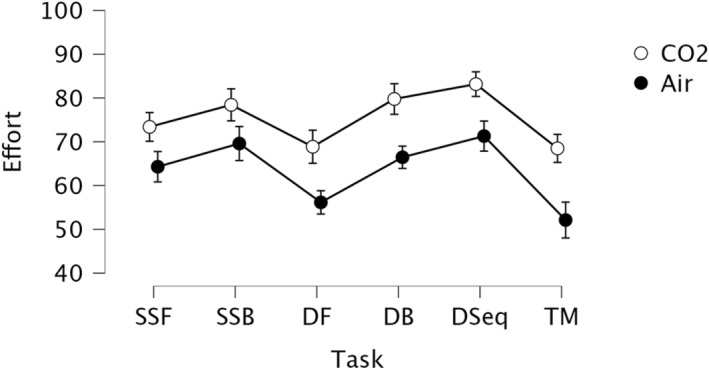
Reported effort ratings for each task completed during CO_2_ inhalation and air inhalation.

Effort ratings are displayed in Figure 3. Repeated measures 2 × 6 ANOVA examined the effect of Inhalation, Task and Inhalation × Task on reported levels of mental effort. Participants reported substantially more effort when completing tasks during the inhalation of CO_2_ versus air, F(1,29) = 8.43, *p* = 0.007. This effect was largest for verbal Digit Span tasks and the Trail Making task and smallest on visual Spatial Span tasks (though the effect of Task × Inhalation was small, *F* < 1). A main effect of Task, F(5,145) = 10.09, *p* < 0.001, reflected greater effort during high load verbal (DB) and spatial (SSB) ‘backwards tasks’ and sequencing (DSeq) tasks.

### 3.4 Processing Efficiency

**FIGURE 4:**
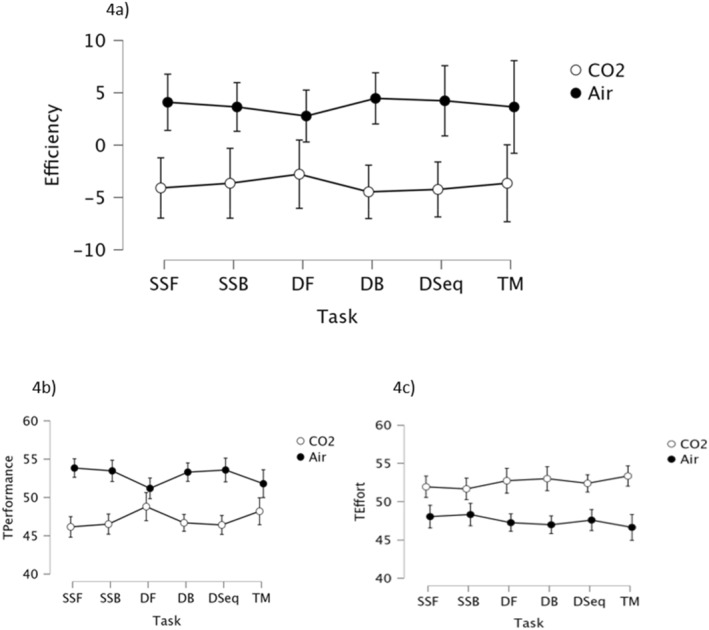
Processing efficiency scores for working memory measures during inhalation of CO_2_ and air (4a). Corresponding standardized (T‐scores) for CO_2_ and air on performance (4b) and effort (4c).

Processing efficiency scores and corresponding T‐scores for performance effectiveness and effort are displayed in Figure 4. A 2 (inhalation) × 6 (task) ANOVA examined the effects of inhalation, task and their interaction on processing efficiency.

CO_2_ inhalation had a negative impact on processing efficiency across tasks, F(5, 145) = 25.763, *p* < 0.001, *n* ^*2*^ = 0.470. CO_2_ impaired processing efficiency on SSF (*d* = 0.86), SSB (*d* = 0.74), DB (*d* = 0.89) and DSeq tasks (*d* = 0.83), but the effects on DF (*d* = 0.56) and TM were small (*d* = 0.29) (Figure 4a). Inspection of Figure 4 and earlier analyses of performance and effort measures show that CO_2_ impaired processing efficiency by both impairing performance effectiveness (Figure 4b) *and* increasing effort (Figure 4c) on SSF, SSB, DB and DSeq tasks, whereas CO_2_ impaired processing efficiency on the DF and TM tasks by increasing effort required to complete the task.

## 4 Discussion

We explored the effects of experimentally‐induced anxiety on clinical neuropsychological assessments of working memory. CO_2_ inhalation produced large increases in subjective anxiety, heart rate and blood pressure. These within‐subjects effects were consistent with those observed in previous 20‐min 7.5% CO_2_ challenge studies (Gillan et al. 2021; Garner et al. 2011, 2012) and supports the use of this method in anxiety induction studies.

### 4.1 Working Memory Performance

During CO_2_ inhalation participants demonstrated poorer working memory *performance*. We observed CO_2_ ‐induced deficits in visuo‐spatial working memory, in both low and high cognitive load conditions, consistent with previous studies that have shown anxiety‐related disruption in spatial WM regardless of task difficulty (K. Vytal et al. 2013). In contrast, the effect of CO_2_ ‐induced anxiety on verbal working memory performance varied according to the cognitive load of the task. Performance on the low‐load digit span forward task was unaffected by CO_2_‐induced anxiety, whereas performance on higher‐load tasks (backwards and sequencing tasks) were impaired during CO_2_ challenge. K. Vytal et al. 2013 observed anxiety‐impairment in low and medium‐load verbal WM n‐back tasks, but not on high load tasks, suggesting high load tasks may consume resources that limit the magnitude and effect of anticipatory (verbally mediated) anxiety during threat of shock on verbal WM performance. It is not possible to compare (across studies) WM load across digit span and n‐back effects, however our results converge to suggest the effects of anxiety on verbal WM appear sensitive to task demands. Consequently, measures of effort and efficiency will prove useful in profiling the general and load‐dependent effects of anxiety on WM function.

### 4.2 Effort and Efficiency

Participants reported substantially more effort when completing all tasks during the inhalation of CO_2_—particularly for high load verbal digit span forward and sequencing tasks. The effect of CO_2_ versus air on effort was largest on ‘low load’ digit forward and the trail making tasks—these tasks required least effort to complete during normal air, and suggests participants may have had capacity to increase effort on these tasks to maximise performance. These results suggest that regardless of modality or task demands, greater mental effort was invested to compensate for the adverse effects of anxiety, consistent with attentional control theory (Derakshan and Eysenck 2009; M. W. Eysenck et al. 2023). The analysis of efficiency scores support this interpretation—during low‐load verbal tasks with minimal central executive involvement, compensatory effort was successful in maintaining performance, but at a cost of reduced efficiency. However, across higher load verbal and both spatial working memory tasks, the investment of additional resources was not sufficient to maintain performance, reflected in particularly poor working memory efficiency during CO_2_‐induced anxiety. This extends previous research on the impact of induced anxiety on mental effort and WM efficiency (Edwards et al. 2015, 2016; Hadwin et al. 2005).

### 4.3 Limitations and Future Research

Our study was powered to examine the effects of CO_2_ inhalation on working memory, but not further interactions with trait anxiety. Our healthy participants were appropriately screened to ensure good physical and mental wellbeing prior to CO_2_ challenge and, consequently, levels of trait anxiety were low across our sample. Future studies should examine whether the effects of acute anxiety (e.g., CO_2_ challenge or situational stress) are moderated by concurrent high levels of trait anxiety and/or depression. K. E. Vytal et al. (2016) found that individuals with clinical anxiety (GAD) under threat (of shock) showed verbal WM impairments (n‐back) on high and low load tasks, whereas low anxious individuals improved their performance on high load tasks during threat. Our study was not designed to covary individual differences in CO_2_ response (self‐report or autonomic response) with WM function—our measurement of subjective anxiety and autonomic arousal at baseline and immediately post‐inhalation prevented time‐course analyses that could link individual differences in response to CO_2_ with WM behavioural measures of effectiveness and efficiency during the 20 min inhalation. Consequently, it remains unclear whether early autonomic effects (see Gnacek et al. 2025) precede subjective experiences of anxiety and greater impairment in WM, and whether perceptible increases in effort further exacerbate subjective and autonomic markers of anxiety. We are mindful that five participants withdrew after completing the CO_2_ inhalation—likely due to experiencing a strong response—and drop‐out rates in mood induction procedures remains a challenge for within‐subject repeated measures designs. We administered the neuropsychological assessments in fixed order to reduce within‐subject variability, but we were not able (within the 20 min inhalations) to include additional clinical neuropsychological measures of executive functioning, such as inhibition (e.g., DKEFS Colour‐Word Interference). Instead, we included self‐report measures of effort to determine measures of processing efficiency. While ‘off‐line’ self‐report measures of effort are likely to be most appropriate (accessible and affordable) in clinical settings, we encourage future studies to validate self‐report effort measures alongside objective ‘on‐line’ measures that are sensitive to the trajectory and peak effects of arousal (Gnacek et al. 2025) and cognitive load (e.g., pupilometry and EEG/evoked potentials; Hepsomali et al. 2019). Finally, it is important to note that cognitive testing was undertaken using in‐person, paper‐based tests rather than digital tools, which may have resulted in administration variability. However, manualised procedures were followed for each test and using clinical tools allows findings to be generalised into clinical settings.

## 5 Conclusion

We observed strong effects of CO_2_‐induced anxiety on WM effectiveness and efficiency. Our results highlight the need to consider anxiety in clinical neuropsychological assessments, particularly in neurological populations where a deficit in working memory is suspected or where anxiety is likely to be elevated for example acute stroke (Grosdemange et al. 2015). In our study the effect of CO_2_ on state anxiety measured by the modified GAD‐7 was greater than the effect on the longer STAI‐S. Consequently, short anxiety screeners (e.g., modified GAD‐7, or single item visual analogue measures) may be a practical but important addition to neuropsychological assessments in clinical practice.

## Author Contributions

**Warren Dunger:** conceptualization, methodology, formal analysis, investigation, writing – original draft, visualization, project administration. **Marc Edwards:** investigation, writing – review and editing, project administration. **Alex Board:** investigation, writing – review and editing, project administration. **Matthew Garner:** conceptualization, methodology, formal analysis, resources, writing – review and editing, supervision.

## Funding

No funding was received for this research. This research was undertaken as part of an educational project for a Doctorate in Clinical Psychology by the lead author.

## Conflicts of Interest

The authors declare no conflicts of interest.

## Data Availability

Data supporting the results of this paper are stored in the University of Southampton data repository and can be requested by contacting the lead author. PURE UUID: dbfd6ee8‐4245‐486d‐824d‐c45fa6f1f12c.
